# Supplementary material for: The in-vitro effect of famotidine on sars-cov-2 proteases and virus replication
Source: Sci Rep. 2021 Mar 8;11:5433. doi: 10.1038/s41598-021-84782-w (PMC7940615; doi:10.1038/s41598-021-84782-w)
Supplement: Supplementary file 1 — Supplementary information. [file 41598_2021_84782_MOESM1_ESM.pdf]

THE IN-VITRO EFFECT OF FAMOTIDINE ON SARS-COV-2 PROTEASES AND VIRUS REPLICATION

Madeline Loffredo[1], Hector Lucero[1], Da-Yuan Chen [3,4], Aoife O'Connell[3,5], Simon Bergqvist[2], Ahmad Munawar[1], Asanga Bandara[1], Steff De Graef [1], Stephen D. Weeks[1], Florian Douam,[3,5], Mohsan Saeed,[3,4], Ali H. Munawar[1*]*

[1] *Bisect Therapeutics, Inc. 45 Dan Road Canton, MA, USA*
[2] *Biofizik, Inc. 9865 Mesa Rim Road, San Diego, CA, USA*
[3] *National Emerging Infectious Diseases Laboratories (NEIDL), Boston University, MA, USA*
[4]*Department of Biochemistry, Boston University School of Medicine, Boston, MA, USA*
[5]*Department of Microbiology, Boston University School of Medicine, Boston, MA, USA*

Supplementary Figures and Tables

Table S1: Summary of the kinetic and equilibrium dissociation constants determined by SPR at 10 ^{o}C. The K_d for *compound 6* and *ML188* were determined from the binding isotherms shown in **Figure 2. B** & **D** (in the main text). Famotidine and *compound 6* were tested up to a maximal dose of 50 mM and *ML188* up to 5 mM. No binding was detected for famotidine to either protein. The K_d for the tool compounds was determined from analysis of the binding isotherms (**B** & **D**). Standard deviation from the mean was determined from triplicate experiments.

Figure S1: Antiviral effect of famotidine on virus yield (plaque formation) in Vero E6 cells. Remdisivir (blue) produced a dose-dependent antiviral effect when tested at a range of 0.1 µM and 62.5 µM concentrations. Famotidine (green) produced no effect on limiting virus plaque formation when tested between the ranges of 0.32 µM and 200 µM. Each point represents the mean ± standard deviation of triplicates.

Figure S2. Antiviral activity of famotidine in (a) Vero E6 and (b) human lung A549 cells assessed by qRT-PCR. Quantitative real-time PCR was used to determine SARS-CoV-2 RNA with primers for the viral envelope (E) gene. Viral mRNA copies/mL of culture medium are shown when infected cells were grown in the presence of a range of famotidine (green) and remdesivir (blue) concentrations. While remdesivir exerts a dose-dependent effect on suppressing virus replication as seen by reduction in viral RNA copies, famotidine showed no effect in either Vero E6 or human lung A549 cells.

Figure S3. Immunofluorescence staining of SARS-CoV-2 infected Vero E6 and human lung A549 cells in the presence of famotidine. Virus infected cells were fixed with paraformaldehyde and stained with an antibody directed to the virus N protein.

Figure S4. Background fluorescence of Famotidine at 355/460 nm and 490/535 nm.
Fluorescence readings for all compounds alone as well as in the presence of substrate were taken at every concentration of compound tested. Shown here are background readings for famotidine taken at wavelengths corresponding to those used for the PL^{pro} and $3CL^{pro}$ *in vitro* inhibition assays. None of the compounds studied in this experiment produced fluorescence of their own at the wavelengths used. All background readings were subtracted from the raw inhibition data to determine final results.

Figure S5. SDS PAGE showing purity of enzymes.
$3CL^{pro}$ and PL^{pro} were expressed and purified as outlined in the materials and methods. SDS PAGE indicates the purity of proteins used in the experiments. $3CL^{pro}$ molecular weight is 33.8 kDa and PL^{pro} molecular weight is 35.6 kDa.

Protein	Ligand	K_a (x 10^5 M^{-1} s^{-1})	k_d (s^{-1})	K_d (μM)
PL^{pro}	Compound 6	2.4 ± 0.9	0.5 ± 0.16	1.9 ± 0.07
PL^{pro}	Famotidine	ND	ND	ND
$3CL^{pro}$	ML188	6.3 ± 0.5	0.2 ± 0.16	0.32 ± 0.06
$3CL^{pro}$	Famotidine	ND	ND	ND

Table S1: Summary of the kinetic and equilibrium dissociation constants determined by SPR at 10 ^{o}C. The K_d for *compound 6* and *ML188* were determined from the binding isotherms shown in **Figure 2. B** & **D** (in the main text). Famotidine and *compound 6* were tested up to a maximal dose of 50 mM and *ML188* up to 5 mM. No binding was detected for famotidine to either protein. The K_d for the tool compounds was determined from analysis of the binding isotherms (**B** & **D**). Standard deviation from the mean was determined from triplicate experiments.

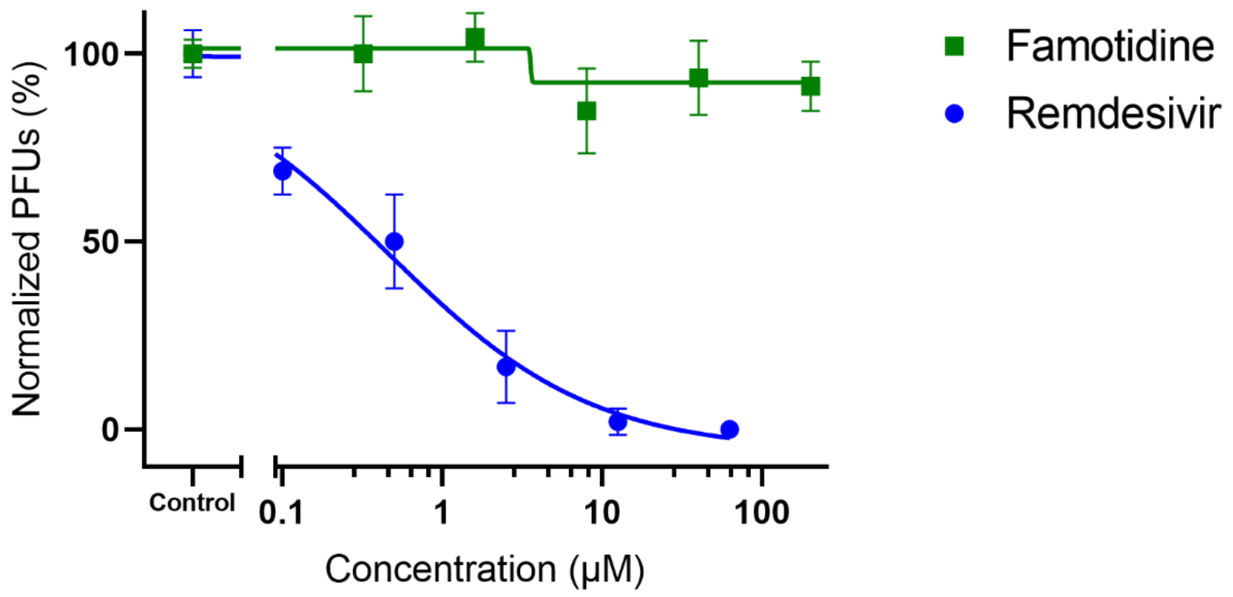

Figure S1: Antiviral effect of famotidine on virus yield (plaque formation) in Vero E6 cells. Remdisivir (blue) produced a dose-dependent antiviral effect when tested at a range of 0.1 µM and 62.5 µM concentrations. Famotidine (green) produced no effect on limiting virus plaque formation when tested between the ranges of 0.32 µM and 200 µM. Each point represents the mean ± standard deviation of triplicates.

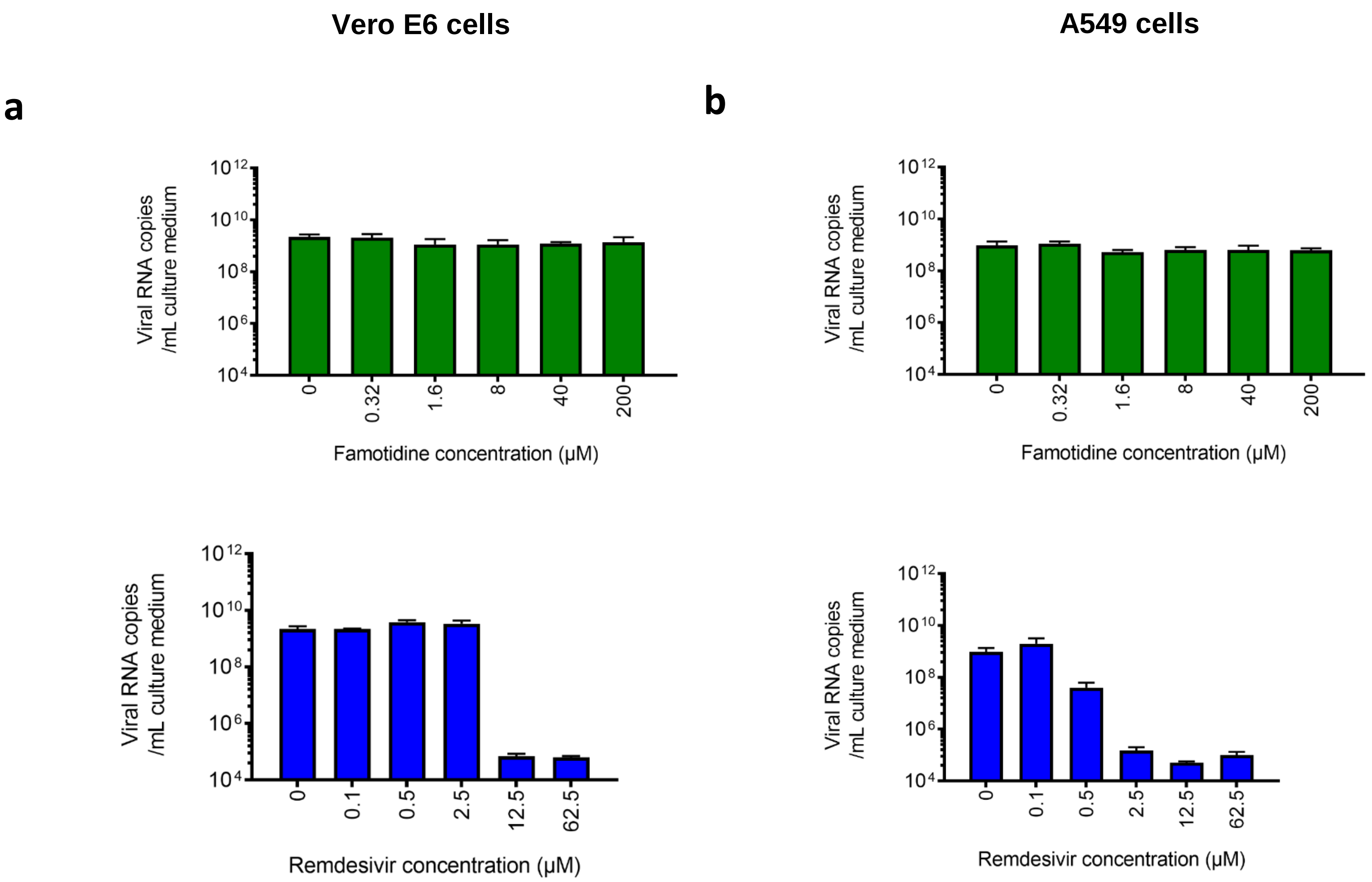

Figure S2. Antiviral activity of famotidine in (a) Vero E6 and (b) human lung A549 cells assessed by qRT-PCR. Quantitative real-time PCR was used to determine SARS-CoV-2 RNA with primers for the viral envelope (E) gene. Viral mRNA copies/mL of culture medium are shown when infected cells were grown in the presence of a range of famotidine (green) and remdesivir (blue) concentrations. While remdesivir exerts a dose-dependent effect on suppressing virus replication as seen by reduction in viral RNA copies, famotidine showed no effect in either Vero E6 or human lung A549 cells.

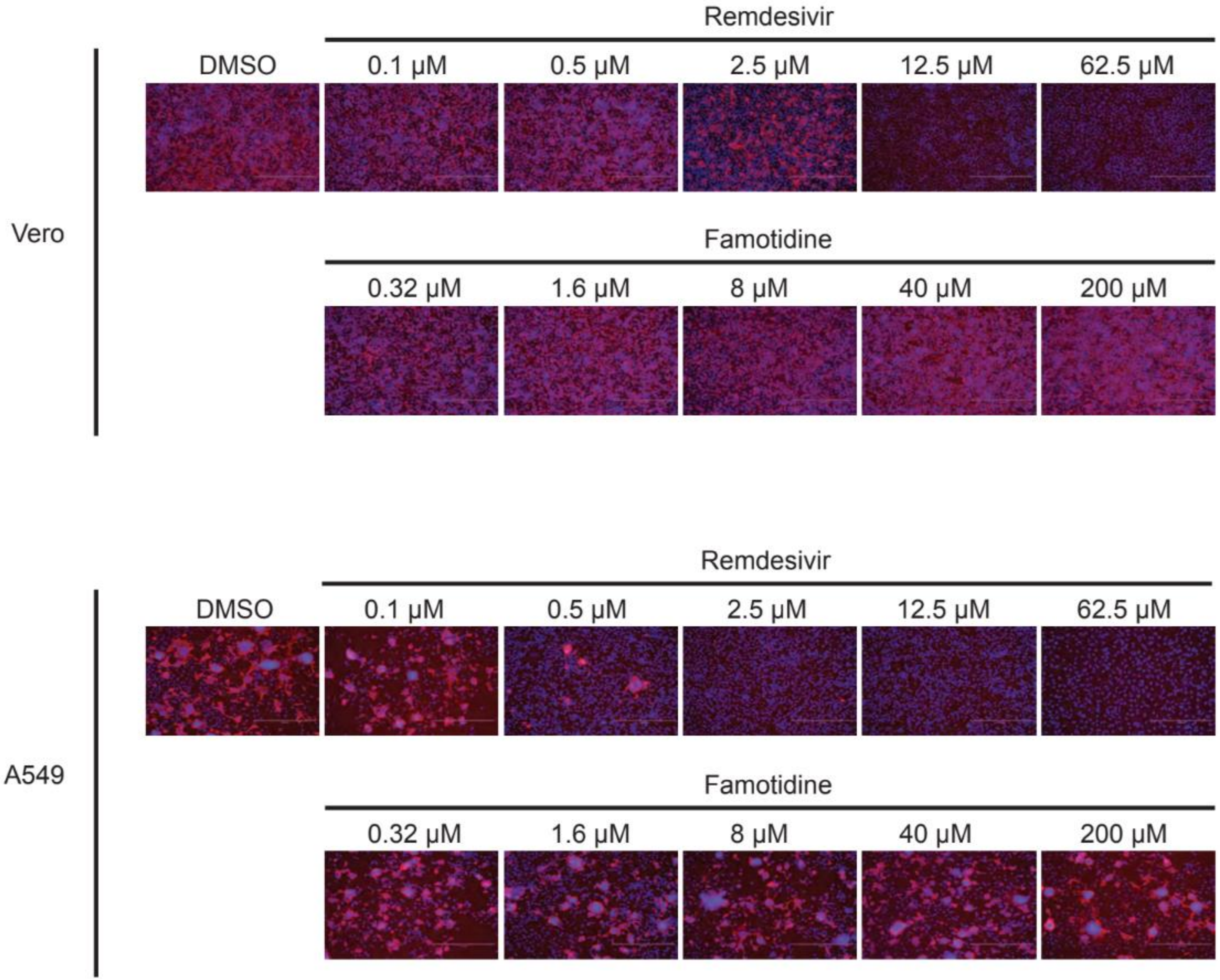

Figure S3. Immunofluorescence staining of SARS-CoV-2 infected Vero E6 and human lung A549 cells in the presence of famotidine. Virus infected cells were fixed with paraformaldehyde and stained with an antibody directed to the virus N protein.

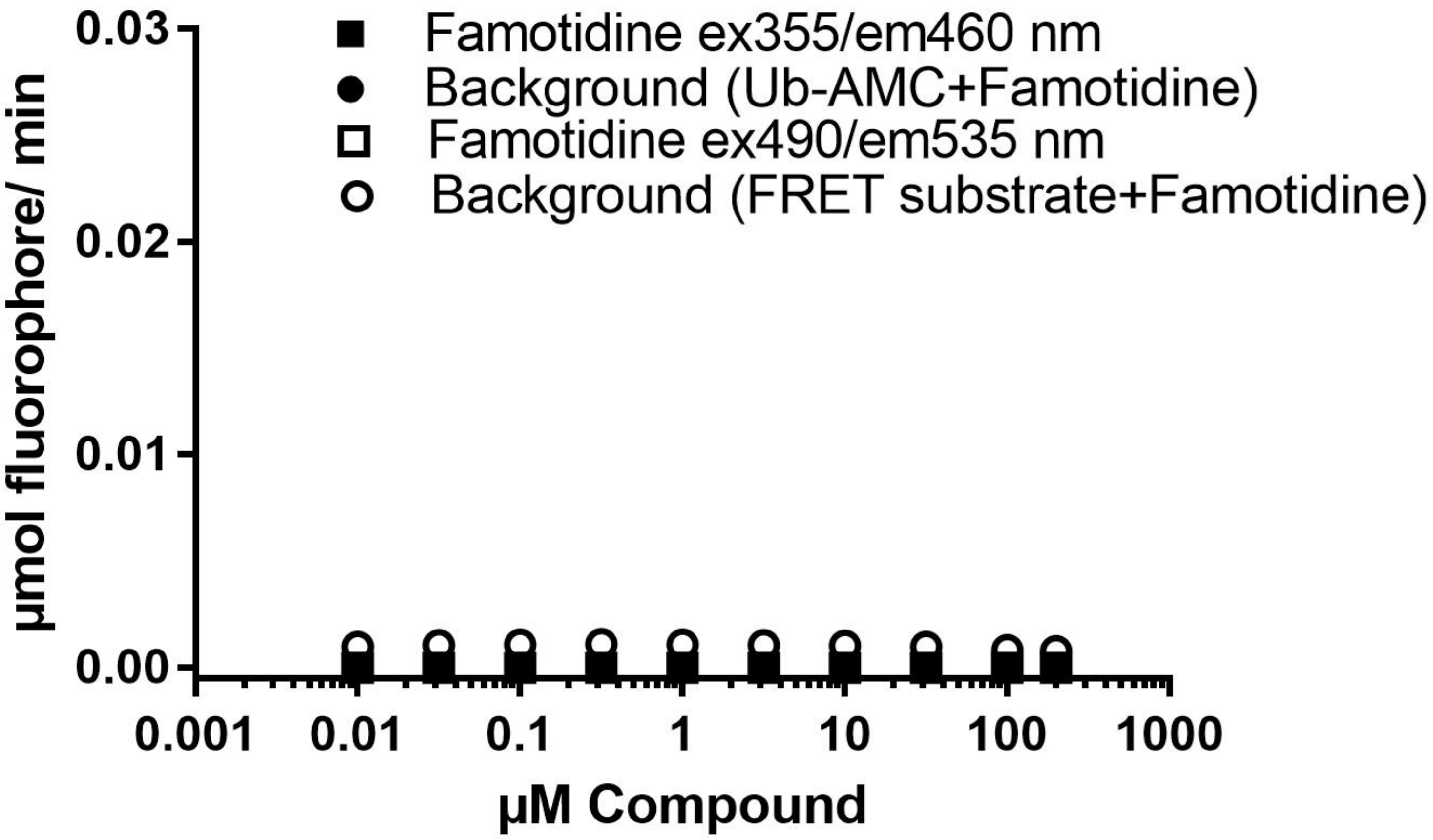

Figure S4. Background fluorescence of Famotidine at 355/460 nm and 490/535 nm. Fluorescence readings for all compounds alone as well as in the presence of substrate were taken at every concentration of compound tested. Shown here are background readings for famotidine taken at wavelengths corresponding to those used for the PL^{pro} and 3CL^{pro} *in vitro* inhibition assays. None of the compounds studied in this experiment produced fluorescence of their own at the wavelengths used. All background readings were subtracted from the raw inhibition data to determine final results.

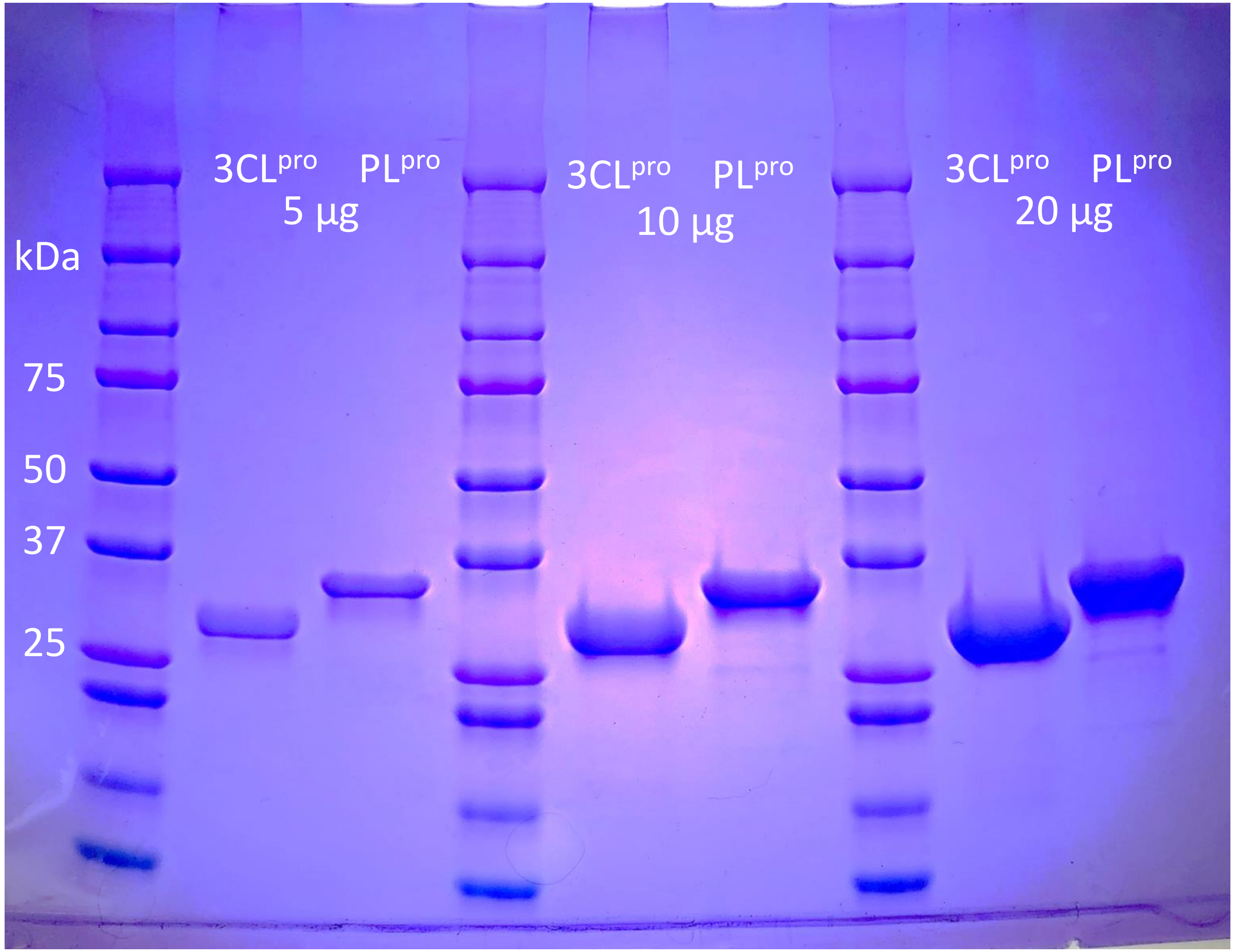

Figure S5. SDS PAGE showing purity of enzymes. 3CL^{pro} and PL^{pro} were expressed and purified as outlined in the materials and methods. SDS PAGE indicates the purity of proteins used in the experiments. 3CL^{pro} molecular weight is 33.8 kDa and PL^{pro} molecular weight is 35.6 kDa.
